# Internet of Things for System Integrity: A Comprehensive Survey on Security, Attacks and Countermeasures for Industrial Applications

**DOI:** 10.3390/s21113654

**Published:** 2021-05-24

**Authors:** Nasr Abosata, Saba Al-Rubaye, Gokhan Inalhan, Christos Emmanouilidis

**Affiliations:** 1 School of Aerospace, Transport and Manufacturing, Cranfield University, Cranfield MK43 0AL, UK; S.alrubaye@cranfield.ac.uk (S.A.); Inalhan@cranfield.ac.uk (G.I.); 2 Faculty of Economics and Business Operations Management, University of Groningen, 9747 AE Groningen, The Netherlands; c.emmanouilidis@rug.nl

**Keywords:** internet of things security, communication protocol, networking, intrusion detection, attacks and countermeasures

## Abstract

The growth of the Internet of Things (IoT) offers numerous opportunities for developing industrial applications such as smart grids, smart cities, smart manufacturers, etc. By utilising these opportunities, businesses engage in creating the Industrial Internet of Things (IIoT). IoT is vulnerable to hacks and, therefore, requires various techniques to achieve the level of security required. Furthermore, the wider implementation of IIoT causes an even greater security risk than its benefits. To provide a roadmap for researchers, this survey discusses the integrity of industrial IoT systems and highlights the existing security approaches for the most significant industrial applications. This paper mainly classifies the attacks and possible security solutions regarding IoT layers architecture. Consequently, each attack is connected to one or more layers of the architecture accompanied by a literature analysis on the various IoT security countermeasures. It further provides a critical analysis of the existing IoT/IIoT solutions based on different security mechanisms, including communications protocols, networking, cryptography and intrusion detection systems. Additionally, there is a discussion of the emerging tools and simulations used for testing and evaluating security mechanisms in IoT applications. Last, this survey outlines several other relevant research issues and challenges for IoT/IIoT security.

## 1. Introduction

IoT-connected devices are predicted to expand to 75 billion by 2025 [1]. Though these devices enhance people’s lives and improve the efficiency of businesses, they also increase the likelihood of vulnerability to attacks from hackers and cybercriminals. IoT technology-enabled devices and components are finding their way into every sphere of work interdependently. A functional disruption to one of these components will severely impact the operations of other interdependent components. There are increasing concerns by experts and policy-makers regarding protecting information and IT infrastructure from these attacks. People, process and technology enterprise constituents are the prime targets of cyber-sabotages. The industrial systems’ security has becomes a paramount concern for all organisations across the industries [2]. Most industrial control systems (ICS) are upgraded legacy systems with connectivity issues that are susceptible to potential attacks. They were not designed for such connectivity, so there is a need to upgrade their security designs. This is being made possible due to the increasing popularity of the Internet of Things, which connects every piece of equipment to the internet to facilitate the communication and management of the pieces of hardware. The result is that increasingly more industrial control systems are interconnected with each other and to the internet, drastically increasing the amount and scale of cyberattack vectors. Key to the successful application of IoT to the industry is to enable real-time monitoring of the network infrastructure and its associate service operations to support the automation of data delivery to achieve secure and high-quality services [3].

**Figure 1:**
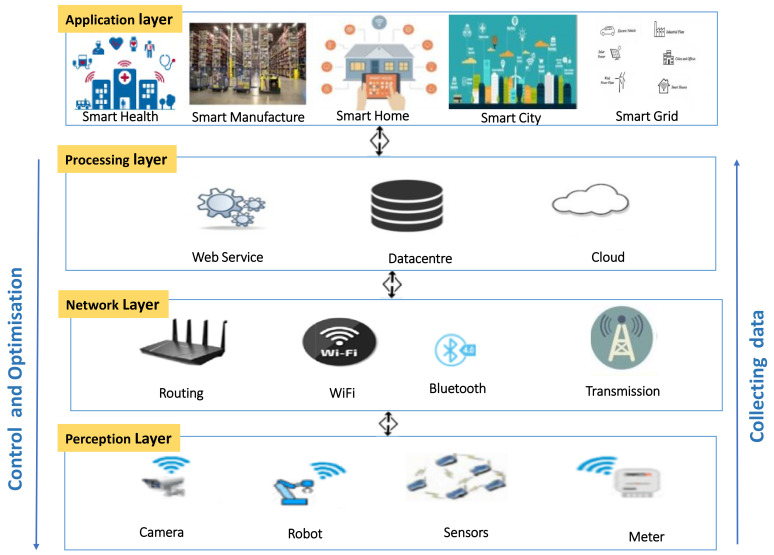
High-level IoT/IIoT architecture.

Figure 1 demonstrates a high-level architecture of IoT and industrial IoT consisting of four components: devices and equipment, networks, cloud and applications. Clearly, as shown in Figure 1, there are four layers: the Perception layer, the Network layer, the Processing Layer and the Application layer [4]. The Perception layer is comprised of numerous kinds of sensors, security cameras, robots, etc. This inventory of machines can be found in an industrial environment, where they could be followed by equipment like industrial robots, automated guided vehicles (AGVs) and other equipment. These devices gather sensory data, track environmental factors and transport raw materials [4,5]. The Network layer may have different kinds of connectivity networks, including WiFi/IEEE 802.15.4, Bluetooth, LoRa, 6LoWPAN and NarrowBand-IoT, and they are responsible for relaying information to the processing systems of the following layer. The industrial Ethernet establishes the base for this layer, which transmits data either to the cloud or to other computers [4,5]. The Processing layer consists of databases and servers and carries out many operations, such as decision-making, refining computation algorithms and storing large volumes of data. The Application layer manages and ensures the meeting of the application-specific needs of the end user. Some significant IoT/IIoT applications include smart homes, smart robots, smart healthcare and vehicle ad-hoc network (VANET), while smart grid, automation and smart factory warehousing are known as IIoT applications [4,5].

IoT and IIoT applications are crucial to the support of services and sensitive data infrastructures. The amount of data generated will increase with applications for healthcare, household and industrial use [6]. According to the authors of [7], approximately 70% of the most frequently used IoT devices are vulnerable to several types of attacks. These attacks include eavesdropping, replay attacks, Denial of Service (DoS)/Distributed DoS (DDoS), sybil and blackhole attacks. For example, in 2016, the DNS provider that supported the internet services and platforms, including PayPal, VISA and Twitter, was attacked by DDoS through the vulnerabilities of IoT devices such as IP cameras, Printers and residential gateways that were infected by malware named Miria [8]. Basic security services such as confidentiality, integrity, authentication, availability and non-repudiation should be appropriate to guarantee IoT protection. However, IoT devices are constrained by their power and memory. For example, objects and devices communicate together in a complicated way by using different security mechanisms. Therefore, security difficulties have become a concern when employing an appropriate security strategy that considers all those limitations. Even though there have been numerous surveys in this field, these surveys, as outlined in the following two sections are limited and primarily focused on either specific IoT security aspect, attacks, layer vision or provide a limited evaluation of the implemented security solutions. This paper aims to present a compact survey in one manuscript evaluating the attacks, countermeasures, simulations and tools, and analysing the security solutions commonly implemented. This paper’s major contributions include the integrity of the industrial IoT systems, which has been highlighted along with a case study discussing the existing security solutions for an important industrial IoT application, the smart grid. Furthermore, the taxonomy of IoT layers, attacks and security countermeasures have been presented, followed by a critical analysis of their practical implementation in previous IoT security solutions focusing specifically on IoT layers. Some of the simulation tools and operating systems (OS) used to investigate security solutions have been presented. Finally, some of the limitations and highlighted recommendations for future work have been discussed.

## 2. Related Surveys and Contribution

Currently, IoT/(IIoT) security and privacy surveys have been quite sparse. The exiting surveys on IIoT system integrity were highlighted in the subsections and followed by surveys on IoT system integrity. Then, how the broad objectives of previous surveys differ from this survey has been clarified.

### 2.1. Surveys on System Integrity for IIoT

The growing popularity of applying IoT in various industries enables the interconnection of anything, anywhere and any time in the industry system context. Several studies have been highlighted industrial IoT privacy, security issues and challenges. Nowadays, embedded devices are commonly used in various domains like vehicles, household appliances, smart grids, aerospace/defence applications, etc. These embedded devices require secure and efficient authenticated encryption that satisfies a variety of resource constraints. The study in [9] presents several lightweight algorithms schemes that can be used for embedded systems for providing integrity and confidentiality. The study compares the impact of different lightweight authenticated encryption schemes in terms of performance, including latency, energy consumption and throughput. Key establishment techniques are one of the countermeasures that ensure IIoT’s security and privacy. A comprehensive study in [10] discussed the symmetric and asymmetric key establishment protocols built at the higher layers and the physical layer; they then outline the importance of developing cross-layer key establishment protocols for the IIoT by examining traditional key establishment protocols. The authors provide an analysis in which the cross-layer architecture allows IIoT devices to create communication keys without the need for a trusted person or the assumption of secret sharing. Another study by Sadeghi et al. [11] proposed a systematic analysis of IIoT systems and their security and privacy challenges, including security threats, attack surfaces and security requirements. They highlight potential solutions towards a holistic security approach to industrial IoT, such as protection design, integrity testing and device management security. A brief history of the industrial internet, its architecture and enabling technologies have been presented in [12]. The study presents various domains for industrial internet application to demonstrate how the integration of industrial internet technologies will transform traditional industries like health care, manufacturing, smart grid and transportation. The study also highlights research challenges and open issues to achieve scalable, safe, secure and interoperable industrial systems. In another work, the authors of [13] detailed some of the vulnerabilities and threats associated with different layers of IIoT. The study also introduces new security challenges and risks to IIoT. Another study in [14] classified the security issues in IIoT systems into two categories: those that apply to both IoT and IIoT, and those that are unique to industrial systems. Finally, the authors of [15] discuss an in-depth overview of Supervisory Control and Data Acquisition (SCADA) system architectures, followed by attacks on the SCADA and outlines the security requirements for SCADA systems. The authors provide a comprehensive analysis of the applied intrusion detection systems on SCADA.

### 2.2. Surveys on IoT System Integrity

There have been numerous surveys conducted on various aspects of IoT systems’ security challenges and proposed solutions. A study in [16] discusses the relevant security solutions of the IoT system model regarding confidentiality, integrity and authentication. The study focuses on security issues related to middleware and mobile devices. Other surveys in [17,18] classifies the security issues of the IoT systems model through specific threats. The study also highlights some of the security solutions’ IoT communication protocol and describes the appropriate protection designed to consider the technological heterogeneity and limited resources of the IoT paradigm. A comprehensive survey in [19] analyses and discusses countermeasures to the possible IoT threats. Individually, the survey evaluates the threats to each layer on the risk assessment in a model-based manner. The survey focuses only on the weakness of the communication protocols, while security issues investigated in [20] examined the IoT protocols included in the Message Authentication Code (MAC) and the physical layer. The study focuses only on the physical and MAC layer communication. The authors of [21,22] discuss a summary of the security challenges associated with the deployment of smart IoT objects. Then, the authors discussed numerous security protocols at the application, transport and network layers, such as Datagram Transport Layer Security (DTLS), Host Identity Protocol (HIP) and Internet Protocol Security IPSec. Moreover, they discuss how the combined cryptography algorithms and the lightweight algorithms utilised in IoT, included in the Elliptic Curve Digital Signature Algorithm (ECDSA) scheme and some of the hash functions, the Rivest–Shamir–Adleman (RSA) scheme, Advanced Encryption Standard (AES) algorithm and the Tiny Encryption Algorithm (TEA) cipher. In [23], a discussion of cryptography algorithms that impact the IoT has been offered. Due to the IoT devices’ resources constrained to memory and processing capabilities, the authors of [24] present a comparison of lightweight algorithms implemented on different software tools and hardware. The improved results of the implemented algorithms change from the application to the software platform. Moreover, they reported that lightweight algorithms are the most crucial aspect used in IoT to enhance security and reduce power and memory consumption. Furthermore, they highlighted a lightweight classification based on their function, which includes hash functions, stream ciphers and block ciphers. A comprehensive survey of IoT’s current vulnerabilities and security threats based on its architecture, communication and application is presented in [25]. The authors also proposed a potential solution to mitigate the weaknesses and threats to the IoT environment. In [26], the authors proposed a survey mainly focused on the security and privacy issues of IoT applications in smart cities. The authors also analysed work that was carried out regarding securing smart cities. The work in [27] classified the IoT security roadmap was based on a cognitive and systematic method. It highlighted the security issues included in (i) data privacy, (ii) trust and (iii) identification and those in the authentication of smart manufacturing as a case study. A comprehensive study is presented in [28] that highlights the vulnerabilities and solutions related to privacy in IoT. On the other hand, the authors of [29] reviewed the characteristics of IoT. Moreover, they discussed DoS/DDoS attacks against the IoT networks and the MAC layer. Additionally, the authors analysed the mechanisms used for mitigating the DoS/DDoS attacks within IoT networks, such as Intrusion Detection Systems (IDSs). The authors of [30] propose a survey highlighting recent IoT security studies from 2016 to 2018. The study discusses the security issues related to encryption, trust and authentication. From the discussion in Section 2, it is clear that much work has been done in this field. However, the studies mentioned above are limited and mainly focused on specific IoT security aspects, attacks, layers, and a limited evaluation of the existing security solutions. Therefore, there is still a need for a compact survey on most existing IoT/IIoT security attacks, solutions and countermeasures. To conclude, this paper will provide the reader with a full picture and understanding of the state-of-the-art IoT/IIoT security attacks, solutions and countermeasures. The paper also highlights some of the simulation tools and operating systems (OS) used to investigate security solutions. Compared with the survey papers discussed in the literature, our significant contributions are highlighted and summarised in Table 1.

The remainder of the paper is organised as follows. Section 2 discusses the background surveys and highlights our contribution with regard to the existing surveys. Section 3 highlights the industrial IoT systems integrity as well as some of the existing studies of industrial application of smart grids. Section 4 presents the IoT layers with their corresponding security attacks and solutions. Section 5 presents the necessary security services and discusses standardised security solutions. Section 6 highlights the test bed tools that are used in IoT research. Section 7 discusses the existing studies’ solutions and provides a critical analysis of these studies. Section 8 presents open research directions, and finally Section 9 concludes the paper.

## 3. Industrial IoT System Integrity

ICS is designed to support industrial processes. Several operations and processes, such as distributed energy resources, water treatment or transportation control, are tracked and managed by these systems. ICS systems are (formally) SCADA (supervisory control and data acquisition). ISC has moved away from proprietary, individual systems linked only to integrated and Internet-based standardised technology that exists in a short period. Furthermore, ICS-based products are most frequently found in various consumer or industrial devices such as routers or cable modems, which typically use commercial off-the-the-shelf software [31]. However, ICS are increasingly vulnerable to cyber-attacks and insider threats in industrial applications. Industrial applications should be protected from system and data integrity attacks in order to improve system security. Threats to systems and information integrity could disrupt critical manufacturing programs, reduce productivity and endanger safety and impact business activities. Industrial IoT is gradually becoming common due to its operational application in different areas [32]. As a result, various security vulnerabilities and threats that arise specifically with IIoT applications have been discovered. In the following subsection, the study addresses the existing solutions based on the smart grid application.

### Case Study on IIoT Application: Smart Grid

The term “Smart Grid” refers to an enhanced energy supply chain that extends from a large power plant to our homes and businesses. The fundamental premise of the smart grid is to enhance the nation’s electrical distribution infrastructure with tracking, review, control and communication capabilities in order to optimise system throughput while minimising energy consumption. The idea of the smart grid is implemented to facilitate its implementation. Despite the many advantages obtained from conventional networks, the smart grid has been exposed to a number of vulnerabilities and attacks [33]. This paper highlights some of the current research being conducted to address the availability, integrity and confidentiality of smart grid infrastructures’ communication and control system. The main security issues facing users of the smart grid connection users are consumer privacy, confidentiality and maintaining the integrity of energy consumption. A security protocol is developed for the smart grid using different double auction mechanisms and homomorphic encryption [34]. This protocol provides authentication, security as well as compatibility to the smart grid technologies. It assigns pseudo-identity to each consumer in the smart grid and ensures the anonymity of consumers during communication. However, the homomorphic encryption schemes in [34,35] generate a lengthy cipher-suit than the plain text, resulting in a large delay for encryption and decryption. A lightweight authentication and key agreement are suggested in [36] for a smart metering network. It exploits hybrid cryptography, i.e., Elliptic Curve Cryptography (ECC), and a symmetric key for providing security. Before applying cryptography, both gateway and smart meters are authenticated mutually. The hybrid cryptography-based security scheme protects against many attacks, but it lacks in focusing on many privacy features, such as unforgeability and undetectability. Several ECC-based authentication schemes [37,38] have been proposed for the smart grid network. However, they do not satisfy all the security features in the smart grid. A pairing scheme between smart meters and server is an expensive operation, and it does not apply to smart meters with low power. In [39], a Certificate-Less Two-Party Authenticated Key Agreement (CL2PAKA) scheme is suggested for smart grid applications. The CL2PAKA does not need to perform any pairing operation, and it implements only four scalar multiplication operations on ECC. The main disadvantage of a certificate-less authentication schemes [39,40] is that the identity information cannot provide a public key for a long time. That means the identity of consumers alone is not sufficient to provide a public and secret key. In [41], a privacy-preserving architecture is suggested for the smart grid using a Q-learning-based optimised approach. It exploits the cryptography technique to outsource multiregional electricity data securely. It implements three dynamic protocols to perform primary operations in Q-learning—Q value updation, Q-learning training and knowledge replaying with encrypted packet information. However, it consumes more time to reach an optimal value, and so it is less applicable to resource-constrained smart grid applications.

It is clear that the existing works mostly apply either symmetric or asymmetric encryption schemes. The former technique has to share the secret key in advance, and the latter does not need to set a shared key in advance. However, the latter technique consumes more computational cost than the former encryption technique. Therefore, it is an effective method to generate a shared key and encrypt the data. For smart grid application, it is important to provide an effective authentication and security scheme in a lightweight manner. As smart meters in advanced metering infrastructure are low energy devices, high complex security schemes are not applicable to smart meters.

## 4. Classification of Security Attacks

**Figure 2:**
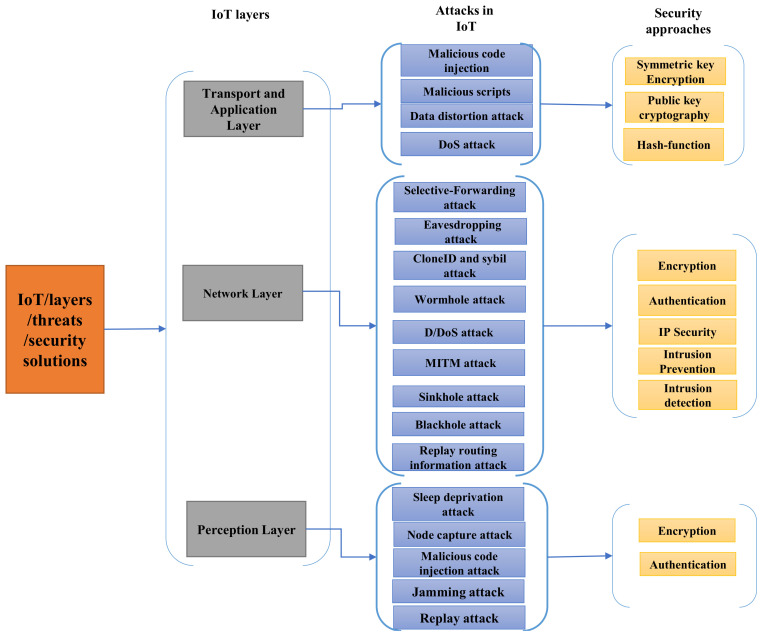
Classification of IoT layers, threats and security approaches.

IoT includes a wide range of devices and equipment ranging from small embedded devices and even advanced large servers. There is a need to highlight the security issues at different IoT layers. Figure 2 shows a classification of IoT security issues along with the security solutions for each of the IoT layers. The most common IoT layer architecture is divided into three layers: perception, network, and the application layer. These three layers contain a large range of information with various enabling features and technologies. Moreover, the following sections present the IoT/IIoT layers with the security attacks of each layer.

### 4.1. Perception Layer with Security Attacks

The perception or device layer includes objects with attached sensors, smart meters, robots, cameras, etc. The perception layer identifies and collects the target sensor data, for example, related to movements, vibrations, chemicals in the atmosphere, heat, orientation, humidity or acceleration. These data are sent to the network layer and then to an information processing system [42].

**Node Capture Attacks:** These attacks involve attackers capturing/replacing a node or modifying hardware, exposing sensitive data for the management of digital rights, such as access/cryptography keys. Replacement nodes may then act maliciously, making the whole IoT network insecure [43].

**Malicious Code Injection Attack:** Malicious codes are injected within the node’s memory via its debug modules. These codes can carry out undesirable activities and may allow an attacker to access the entire network. Attacks generally occur when upgrading software/firmware through over-the-air (OTA). When devices are in the operating mode (e.g., during a scheduled firmware update), an intruder may insert Trojans into the machine (requires device reboot). The security challenge, in this case, is divided into two categories: proper authentication, identification of an edge device in the network and making sure that drivers or malware are not installed on peripheral devices in the name of updates and upgrades [43].

**Sleep Deprivation Attack:** Sleep deprivation attacks resemble denial-of-service attacks, as they drain the edge device’s batteries, while these devices are usually intended for low-power operation. Consumption is increased by making hardware modifications or injecting infinitely-looping codes into the memory [43].

**Jamming attack:** These attacks interfere with the tag reader air interface, disrupting communications or altering communications. Jamming is done through a long-distance, powerful transmitter or passively, for example, through shielding, which can succeed due to the sensitivity of the interface. RFID systems can be jammed through radio noise which matches the system’s frequency [44].

**Replay attack:** This type of attack involves repeating of an authentication code used by an authorised individual, either by cloning the authorised tag or sending a signal again after eavesdropping on signals sent by a device with the correct antenna and card. Replay attacks require specific data which the tag sends in communications [44].

### 4.2. Network Layer with Security Attacks

The network layer is responsible for transmitting data to and from various things or applications through interfaces or gateways between heterogeneous networks and via a variety of communication technologies and protocols. The network layer receives the processed data from the perception layer and determines the routes for transmitting it to the IoT devices, hub and gateway through integrated networks [45].

**Selective-Forwarding Attacks:** These attacks are DoS attacks. Only selected packets are forwarded by malicious nodes and aim to disrupt the route of the path, although any protocol could be targeted. Thus, attackers might forward every RPL control message while dropping all other packets. When combined with a sinkhole or other attacks, selective forwarding can be highly damaging [45].

**Eavesdropping attack:** This type of attack relies on the signal sent out by RFID tags when required by RFID readers, through eavesdropping on this signal being sent to an RFID reader with authorisation to identify the frequency and tag group used. Clear text is used by the majority of RFIDs based on cost and memory limitations, and this allows the eavesdropping to succeed [44].

**Clone ID and Sybil Attacks:** Clone ID attacks involve copying a legitimate node’s identity to a second node for reasons such as accessing a greater proportion of a network or counteracting vote schemes. Sybil attacks utilise a number of logical entities for one physical node to control an extensive network area with no need for more nodes to be used [45].

**Wormhole Attack** These attacks generally target traffic flows and network typologies. Wormhole attacks are performed by producing a tunnel that links two attackers for the selective transmission of traffic via this route [46].

**Denial of Service (DoS):** In denial of service or DoS attacks, a targeted network/ computation source is disrupted, potentially reducing capacity on the network. For IoTs, DoS attacks can be Distributed Denial of Service (DDoS) or a simple DoS attack. The simple attack needs a tool for sending packets to crash or restart a system/network, while DDoS may use one attacker with less force than a proxy. These attacks can disrupt and prevent access to networks [47].

**Man in the Middle attack:** These attacks involves interception and alteration of node-to-node communications, using a range of strategies. After the node–node link is interrupted and the data altered in real-time, it can then be monitored by the attackers [48].

**Sinkhole Attack:** Sinkhole attacks compromise nodes within a network and uses these to transmit false routing data to adjacent nodes, claiming to have the shortest route to the base and then dropping or modifying packets routed through them [49].

**Blackhole Attack:** These attacks involve a malicious node placed within the network quietly dropping every packet routed through it by the network, with nothing being passed on [46].

**Spoofed, Alter, Replay Routing Information:** Mutual direct attacks involve spoofing, altering and replaying routing targeting routing data in node-to-node exchanges. Spoofing attacks utilise issues due to the ability to detect an IoT device within a system, for example, by producing a fake error message or producing a routing loop [47].

### 4.3. Application Layer with Security Attacks

The final layer in this novel architecture design is the application layer. This layer formats and presents data and delivers a range of applications to diverse types of users, defining different smart applications for the use of IoT, including smart health, homes, cities, industries and transportation. This layer provides the user with a particular application based on object sensor data [45]. Security is a central challenge in this layer, with frequent issues arising, including the following.

**Malicious Code Injection:** These attacks exploit coding within the software, which damages systems or leads to other unwanted impacts and can avoid detection by anti-virus applications. The code may be self-activating or activated when the user takes a specific action [48].

**Malicious Scripts:** these involve networks or IoT devices that are connected to the Internet. The attack is carried out by running malicious codes or x-scripts which look like legitimate scripts and which the user must access, to be data theft and systems failure [50].

**Data distortion attack:** these uses code within the software to damage systems or lead to other unwanted impacts and avoid detection by anti-virus applications. The code may be self-activating or activated when the user takes a specific action [50].

## 5. Connectivity and System Integrity Approaches

The IoT allows devices to share and transfer data among users and devices to accomplish specific goals. Therefore, security is intrinsic to the deployment of IoT due to the sensitivity and connectivity of applications, such as military and defence, smart homes, healthcare, and railway systems [16,51]. The following are the necessary security services in IoT and industrial applications.

**Confidentiality:** It is crucial to ensure that the messages are secure and accessible to only authorised objects because an intruder could catch the data flow between sender and receiver, and confidential data could be exposed. Moreover, these data should be unknown to intermediate users. In IoT, an object could be machines, devices, sensors, and internal and external objects. For example, it is required in IoT that the end-to-end message is secret. Furthermore, the data stored in the IoT device should be unobserved from unauthorised users. Data confidentiality services are supported through mechanisms such as encryption and decryption [52,53].

**Integrity:** It is important to ensure that data exchange between multiple IoT devices is accurate. This means that the data comes from the right source and ensures the data is not modified during the transmission [54]. For instance, the stored data of medical patient should not be modified. The most useful protection for providing the integrity service is Message Integrity Codes (MIC) and the hash function mechanism. Furthermore, maintaining end-to-end security communication in IoT is crucial for integrity feature [55].

**Authentication:** It ensures that the data have sent from the right device and not modified during the transmission. In communication, each object should be able to identify as well as authenticate each other. Nevertheless, this process is very challenging due to the nature of IoT; numerous objects included in people, devices, processing units, and services providers may be required to communicate with each other. Due to these requirements, the authentication mechanism in every communication within IoT is necessary [56].

**Availability:** The vision behind IoT is to link as many devices as possible. Availability ensures that all the data are available to the objects whenever they are needed. Nevertheless, the data component is used in IoT, and devices and services should be available and reachable whenever needed [57]. Besides, firewalls and IDSs are the most effective security mechanisms which can be used to detect malicious activity and intrusions to ensure the availability of services [58].

**Lightweight solutions:** Lightweight security is considered a unique feature due to the power capabilities and computational speed of the IoT devices. A lightweight solution should be considered through designing and executing protocols in the encryption and authentication of IoT devices. Meanwhile, these algorithms can run with limited capabilities on IoT devices [17,59].

**Replay protection:** A stored data packet in the intermediate node can be compromised and replayed back later. The replayed data can comprise a sensor reading, for example, temperature reading or blockchain transaction. It is crucial to provide a mechanism to detect replay or duplicate messages. This can be accomplished through the nonce mechanism, integrity-protected sequence number, or timestamp [60].

### 5.1. Communication Security

The communication in IoT must be preserved by the designated security mechanism discussed in Section 5. Security can be provided at different layers by using the combined Internet security solutions. Generally, the protection of the IoT communication can be delivered through end-to-end security or intermediate devices [20]. The standardised IoT stack with the standardised security solution at IoT layers is shown in Table 2.

**IEE802.15.4 security: Link-Layer level**

The IEEE 802.15.4 protocol is widely used for short-range communication in the IoT environment. It is specifically responsible for information transmission at the physical and MAC layers. IEEE802.14.5 protocol is used as link-layer security in communication networks such as IPv6 over Low-Power Wireless Personal Area Networks (6LoWPAN). The link-layer provides secure communication on hop-by-hop to all the nodes in the network. The specification of 802.15.4 outlines different security sets that can be included in encryption and authentication (AES-CCM), encryption only (AES-CTR), authentication only (AES-CBC- MAC), and no security. All the communication is protected through the pre-shared key. The shared key must be secured because if the attacker gains the key, the attacker can compromise the whole network [18]. Although the IEEE 802.15.4 protocol provides critical security services, it does have some limitations. More precisely, it is incapable of safeguarding the privacy and confidentiality of acknowledgement messages (ACK).

**LoRaWan Security**

The long-range wireless area network (LoRaWan) was designed to enhance the functionality of Low-Power Wide-Area Networks (LPWANs) in terms of power consumption, storage, long-range communication and transmission cost. The four critical components of its architecture are end nodes, gateways, network and applications servers. End nodes are typically Internet of Things devices that gather data from their physical environment and transmit them to gateways through the LoRa physical layer. The gateways then transmit this data to a network server. This can be carried out through IEEE 802.11 (Wi-Fi), satellite, IEEE 802.3 (Ethernet) or other systems. The network server is responsible for data control by conducting necessary security operations and scanning for duplicate packets. It then sends the information to application servers, which function as the foundation for software applications [61]. Two layers of encryption are used in LoRaWan technology. The first security layer is responsible for authenticating the data on the end nodes. This process is performed between the end nodes and the network server using an AES-CTR 128 secret key called the network session key. On the other hand, the second layer ensures that end nodes’ privacy is protected by using an AES-CTR 128 secret key called the application key between the end nodes and the application servers. As a consequence, the LoRaWaN technology’s protection of these keys is important. If any secret key is compromised, a potential attacker would have access to and alter the data. Additionally, when communicating between end nodes and gateways, it is worth remembering that the length of the payload remains constant before and after encryption. An attacker can exploit this by decryption the encrypted messages and recovering the network session key [62].

**IPsec: Network level**

IPsec provides security for the network layer. IPsec provides end-to-end security, which contains a number of protocols such as Encapsulating Security Payload (ESP), which ensures confidentiality and integrity, and the authentication header (AH), which ensures authentication and integrity [63]. IPsec can be used with the IP-based protocols and a transport layer such as User Datagram Protocol (UDP), Transmission Control Protocol (TCP), Constrained Application Protocol (CoAP) and Hypertext Transfer Protocol (HTTP). IPsec is considered the most appropriate for end-to-end security for IoT because of the default security policies that run on a constrained device [64]. Virtual Private Network (VPN) technology is also considered the first line of protection for IoT networks. It enables establishing a private and secure tunnel between communicating parties to safeguard the exchanged data against tampering and passive and active intruders. Indeed, the tunnel construction process begins with establishing a preparatory encrypted and stable tunnel, and then the encryption keys and parameters are negotiated from inside that channel [65]. Besides IPSec and VPN, RPL provides security mechanisms to control messages. Even though it is optional, the integrity of the authentication process and confidentiality of the control message is guaranteed [29].

**CoAP: Transport Layer**

As mentioned above, in addition to the IPsec datagram, the Secure Socket Layer (SSL) or the transport layer security (TLS) is the most commonly used security protocol in web protocols that run over TCP. Still, they are not efficient for use in communication with smart objects in low-power wireless networks [29]. Another version of TLS is Datagram Transport Layer Security (DTLS), which runs over UDP. DTLS provides integrity, authentication and confidentiality solutions. Besides, DTLS provides end-to-end security between applications and transport layers. CoAP is an IoT web protocol, and DTLS is used as a security solution for CoAP protocol [66,67].

### 5.2. Network Security

The network layer’s main task is addressing and routing data packets to integrate countless devices into a single collaborative network seamlessly. The most common network layer protocols include IPv4/IPv6, low-power and lossy (RPL) and 6LoWPAN networks. Although the integrity and confidentiality services secure the messages within the communication, several types of attacks are likely to be accrued on the networks, significantly disrupting the availability of the security services [68]. Eavesdropping attacks can analyse traffic transmitted over a network and affect the privacy of data. Therefore, traffic is then vulnerable to various attacks, such as DoS, Man-in-the-Middle and illegal access attacks. The DoS attacks can be launched to disrupt the networks. Furthermore, the network layer of IoT is highly vulnerable to a MITM attack. The security of the communication will be exposed if the attacker gains access to the keying of the devices [69]. However, protecting the network in IoT is crucial in order to secure services such as integrity, availability and confidentiality when transferring the information at the network layer. firewalls, Intrusion Detection Systems (IDSs), and key management can be employed to secure the network against the routing attacks such as sybil, rankhole, blackhole, clonID, etc. [70]. 6LoWPAN networks are susceptible to attacks from both inside and outside the network. As discussed above, some security solutions can protect the 6LoWPAN, such as IPsec, 802.14.5 and the DTLS. Further to this, RPL networks are also vulnerable to routing attacks aimed at disrupting the network. The specification of RPL defines various security modes: “unsecured” RPL control messages; “pre-installed” RPL, in which nodes have been configured with a symmetric key to generate a secured RPL message; and third, “authenticated”, which are used to operate the device as a router. Or, finally, when a device joins the network using both the pre-installed mode and the preconfigured key, every RPL message has a protected variant, as well as AES/CCM procedures, which are utilised to support integrity and confidentiality [18,71].

### 5.3. Application Security

There are numerous challenges to the application’s security because of the absence of standards that control the communication and the development process of applications. For instance, identity authentication and access permissions can be a reason for concern. There are different IoT applications such as smart home, smart healthcare, smart city, etc. For example, the smart home application can provide the air conditioning and temperature measurement to the client requesting such information. For applications of different purpose for customers with widely varying needs, it is challenging to accomplish authentication and access permissions [6,51]. Data security is a major factor in the application layer. In order to guarantee access privileges and data usage, data encryption and distortion technologies are mostly used in the protection of the privacy of data. Furthermore, recovery mechanisms and data backup must be implemented appropriately, as well as techniques to protect data privacy should be properly selected, such as DNS, TLS, SSL, etc. [72,73].

## 6. Network Simulations and Operating Systems

Designing, developing and evaluating new IoT products and protocols prior to being deployed on a specific environment demands testing and assessment using various tools. For instance, prototyping may not be widespread when using a large number of device nodes during the original exploratory design and evaluation phases, and this is because of the economic and operational restrictions. This is especially the case when the reliability and utility of the protocol under consideration have not yet been demonstrated. Additionally, creating reliable, repeatable experiences that include real hardware can be complicated and often require specific expertise and field knowledge. Thus, some simulators and real operating systems (OS) offer a better choice for setting up reliable scenarios and experiments. However, as mentioned earlier, testing, analysing and evaluating a real test bed are costly and challenging. Therefore, simulators offer high accuracy for scenarios involving heterogeneous elements, energy efficiency, scalability and low-cost [74]. This section examines several simulators and operating systems that use in IoT research.

### 6.1. Simulators

The IoT paradigm predates network protocol research, and many previously available tools for WSN or basic networking research have been modified to include IoT-specific elements. A comprehensive survey conducted in [75] highlighted approximately 21 open-source that have been used in IoT/IIoT. However, this paper mainly focuses on the widespread use of these open-source simulators by academic and industrial user groups, including Cooja, OMNeT++, NS-3 and QualNet3 SCADAsim.

**Cooja** is a simulator configured for emulating a network with sensor nodes and supports several sensor motes included in Wismote, Sky, and Z1 motes, etc. Cooja enables synchronised simulations in three levels: machine code instruction, operating system and application level. Therefore, the majority of protocols and standards implemented through Contiki [76].

**OMNeT++** is a network simulator for modelling communication networks, distributed and other parallel systems, or multiprocessors. OMNeT++ is open-source, discrete-event, C++-based and can be used by education, academic and commercial institutions for simulating distributed systems and computer networks [77].

**NS-3** is a new open-source simulator that was developed to replace the old simulator NS-2. It is a powerful tool for supporting network optimisation and modelling, including internet-stack implementation module TCP/UDP/IPv4/IPv6 stack and NetDevice operations such as IEEE802.15.4, CSMA and WiFi. It also supports the 6LoWPAN stack [78].

**QualNet** is a commercial simulation tool introduced by Scalable Network Technologies (SNT). It is a network software for large, distributed applications and heterogeneous networks. It has an additional extension sensor network library that can be used for IoT specific simulation, which supports the IEEE 802.15.4 networks [74,79].

**SCADASim** is open-source software that aims to provide a framework for rapidly developing flexible SCADA system simulations. SCADASim is a discrete event simulation engine built on top of OMNET++. The SCADASim architecture comprises three primary components: SSScheduler, a real-time scheduler; SSGate, a communication port that implements protocols for communication with the external environment; and SSProxy, a simulation object that simulates an external component within the simulation environment. Additionally, it includes several tools for developing network typologies (the NED language and editor) and a plug-in extension architecture. Plug-ins allow for customisation of the default simulation engine’s actions. For instance, a simulation can be enhanced with a different message scheduler, thus altering the default behaviour of message scheduling [80].

### 6.2. Operating Systems (OSs)

The IoT OS is well suited to IoT devices with low to moderate resource constraints. Small IoT devices are highly specialised devices that place a strict restriction on IoT operating systems to be extremely hardware-specific with minimal capabilities. Medium-sized IoT devices allow the inclusion of a full IP suite and various applications to run on top of the network stack. Additionally, the devices have additional capabilities and can act as servers, hosts or internet routers. Many aspects of OSs were designed for the IoT environment. However, this paper highlights some of these OSs, including TinyOS, Contiki OS, Riot OS, Raspbian, mbeds and Zephyr. However, based on the literature analysis, the most widely used OSs are Contiki OS and TinyOS.

**TinyOS**, designed for Wireless Sensor Networks (WSNs), had been the most popular operating system for public research for many years. Nevertheless, it has not been used recently by researchers because of inactive development. TinyOS is an open-source OS designed for wireless devices that are embedded and have low-power. It has a programming language based on C called NESC (Network embedded system C). It also provides several hardware platforms and supports the 6LoWPAN protocol through IPv6 stack [81].

**ContikiOS** is open-source and the most common operating system used for programming IoT sensor nodes. Furthermore, it is used for multi-tasking and making the network more efficient in terms of memory, wireless sensor systems and networks [74]. In addition to this, Contiki supports IP connectivity for both IPv4 and IPv6. Contiki was developed by a group led by Adam Dunkels at the Swedish Institute of Computer Science [82]. Contiki supports many mechanisms and protocols, for example, RPL routing, application protocol CoAP and MQTT, and 6LoWPAN header compression. Furthermore, Contiki provides a power profiling mechanism named Powertrace, which keeps track of, and estimates the, energy consumed by each sensor node [83].

**Riot** is a free, open-source operating system created by a grassroots group of businesses, academics and enthusiasts and is distributed worldwide. RIOT OS is compatible with ARM Cortex-M3 and Cortex-M4 processors and ARM7, AVR Atmega and MSP430 devices. This OS was written in C and C++ and is distributed under the LGPL v2.1 licence. Gcc, valgrind and gdb are specific development tools that can be part of an SDK included with RIOT OS. Additionally, the SDK architecture is compatible with C and C++ application programming. RIOT is compatible with the vast majority of low-power IoT devices and micro-controller architectures. RIOT OS is compatible with most common communication and networking protocols, including IPv6, TCP, UDP, CoAP, 6LoWPAN and RPL [84].

**Raspbian** is the most common operating system used by researchers in IoT and IIoT projects. It is a low-power, highly extendable, cheap and small board developed by the Raspberry Foundation in the UK to be used for teaching and experimental projects. It runs over a variety of Linux distributions and its primary Raspbian Debian-based operating system. There are different varieties of Raspberry Pi available such as the Pi 3 Model B, Pi 2 Model B, Pi 1 Model A=, B+ and Pi Zero. The latest Pi 4 Model B launched in June 2019; it has 1–4 GB RAM, uses a 1.5 GHz Quad-Core ARM Cortex-A72 CPU, integrated Bluetooth 5.0 and 802.11n wireless LAN [85].

**MbedOS** is a 32-bit ARM cortex-m micro-controller operating system developed by ARM in collaboration with its technological partners ([29]). mbed OS is an open-source operating system that can be used on a wide range of products, from small internet-connected devices to smart cities and smart applications. The entire situation The operating system is written in C and C++. The Apache License 2.0 governs this open-source operating system. When compared to Microsoft’s or Google’s offerings. Device and Component Support with real-time software execution and ease of use by any client are some of the key features of the mbed OS, as are end-to-end Security and an extensive collection of drivers and support libraries [84].

**Zephyr** is a real-time operating system designed for Internet of Things (IoT) applications that the Linux foundation backs. It is common among IoT experts due to the ease with which it integrates with various IoT architectures. Zephyr’s interconnectivity technology is one of the most distinguishing features. It is a library-based operating system with stable memory security and a highly configurable, customised open-source IoT operating system that supports device trees. 8 kb RAM and 512 kb ROM are needed to run this operating system. It includes a device development kit with comprehensive documentation, a comprehensive set of kernel services, non-volatile storage, and virtual file support, among other features [86].

## 7. Security Solutions for IoT and Industrial Systems

### 7.1. Communication Layer Security Solutions

Securing end-to-end communication in an IoT network is crucial. Compressed IP security is proposed in [64] to enable-end to-end security within communications between the traditional internet and the sensor network. Their security approach involves the Encapsulation Security Payload (ESP) and the Authentication Header (AH). It shows that their compressed IPsec can check the integrity of messages by encrypting and authenticating using standardised IPv6 mechanisms. Moreover, they extended their work in [87] detailing ESP for IPsec/6LoWPAN, and then compared its solution with employing link-layer security for IEEE802.15.4. The IPsec/6LoWPAN security and solution’s performance evaluation testbed is built into IEEE 802.15.4, which re-utilised the crypto device through the actual IEEE 802.15.4 transceivers for IPSec /6LoWPAN. Although the IPsec is better than link-layer security for enhancing security and performs better according to the response time, the IPsec consumes more energy than link-layer security. The authors of [88] proposed a security analysis, access control improvements and authentication for the IoT. They offered a practical protocol for IoT by breaking down current access control and authentication approaches. An efficient, simple and secure key function based on ECC, the authentication protocol is utilised to enhance device authentication. A Role-Based Access Control (RBAC) is proposed for the access control rule on IoT network applications. However, the practical implementation of the proposed security valuation was not performed, and communication overhead for the IoT sensor nodes was high.

### 7.2. Network Routing Security Solutions

Although enabling the encryption and the authentication with messages, IoT networks were still vulnerable to many attacks such as sybil, the black-hole attack, sinkhole, fragmentation attacks, selective-forwarding and the “man in the middle” attack. It is crucial to design a system to detect these attacks. Many studies implemented intrusion detection systems against attacks in WSN; however, some research performed IDS against an attack in IoT. IDSs could be placed at every node of Low-power and Lossy Networks (LLN). Due to the nodes being resources constrained, the IDS’s deployed in each physical object must be optimised. The author of [89] have performed a lightweight intrusion detection against malicious attack (DoS), which observes the energy consumption of the node when discovering intrusions. They evaluate the impact of IDS on the sensor node’s energy consumption during the attack, which revealed that higher energy was consumed during the attack observed. However, the author does not show the detection accuracy of their IDS, and they only use one metric and one type of attack. Due to the resource-constrained memory size and computing power, the authors of [90] have proposed lightweight IDSs based on malicious pattern detection. They evaluated their detection in terms of energy consumption and execution time based on two schemes: early detection and auxiliary shifting to decrease the number of matches required for discovering attacks. Although their approach enhances the performance and accuracy faster than the Wu-Manber algorithm, the overhead of auxiliary skipping (AS) was high due to the increase of the pattern. Another distributed IDS architecture proposed in [91] called INTI (Intrusion detection for sinkhole attacks over 6LoWPAN for Internet of Things) for sinkhole attacks. Their solution combined the approaches of “watchdog” and “reputation and trust” for discovering and reducing attacks. INTI contains four roles. First, nodes are categorised as a leader, in which the leader nodes receive each node’s information state. Then, each node monitors the routing traffic. The third is attacker detection. The last nodes are for isolating the attacker. The authors have not analysed the impact of their solution on the resource-constrained nodes in terms of energy consumption. Although they achieved a lower false-positive rate than in [92], it is still high (29%). A hybrid intrusion detection system was proposed in [92] for IoT was called SVELTE. SVELTE targets selective forwarding and sinkhole attacks. SVELTE consists of the main IDS modules, a 6LoWPAN mapper and a mini-firewall at the constrained devices and border router. Based on the requests sent by the border router to the client nodes, the client node responds with information such as ID, parent ID and rank. SVELTE compares the collected information to find any malicious traffic and notifying the border router. They claimed that their proposed intrusion detection could be applied to anomaly detection techniques. The best IDS result is to get a low false positive, low false negative and high true positive. The SVELTE recorded a 90% true positive; however, the true positive rate decreases with the increase of nodes, and there is a high false alarm rate (38%) during the detection of malicious. In a similar study to SVELTE, the authors in [93] proposed network-based ID’s that targeted wormhole attacks. Their IDS measures the collected, received signal strength to identify the suspicious nodes. The results show that the true-positive rate was 90% when the network size was small (eight nodes); however, there a significant decrease with the increase in the network size. Furthermore, the authors only evaluated the impact of their IDS in only one metric and one attack. IDS specification-based was proposed in [94] for IoT. The proposed IDS works against repair and rank attacks. They also designed and implemented distributed architecture to monitor all the nodes over an RPL limited state machine. Even though they claimed that their proposed system could successfully detect routing threats with a sensible overhead, the authors did not implement IDS and did not specify the form of communication among monitoring nodes. Due to all monitoring nodes need to store the ranks, preferred neighbouring nodes, and IDs. The finite state machine was implemented on all monitoring nodes with an anomaly state to discover the attacks if the monitoring nodes could not observe whether the node is not an attacker. Further to this, the same authors [95] implemented an IDS specification-based which targets rank, local repairs, sinkhole, DIS and neighbour attacks. They employed a group monitoring structure, where the network is split into multiple clusters. The IDS is located at the head of each cluster to monitor the cluster based on the configured role to reduce computation and storage. The head cluster sends requests periodically to all cluster members; each member responds with parent information, neighbour lists, and rank information. Their result shows that IDS can be detected more effectively than network attacks with a slight amount of overhead.

The authors of [96] propose an efficient, secure route optimisation protocol for the Proxy Mobile IPv6 (PMIPv6). The presented protocol enhanced the existing routing protocol (PMIPv6) when using security, specifically when using authentication, complete forward secrecy, key exchange and privacy when supporting the protocol mutual. Their approach provided secure transmission and reduced packet loss, latency and delay. The authors of [97] proposed a novel trust mechanism which was implemented in a test bed experiment based on the SecTrust-RPL routing protocol. The proposed protocol SecTrust-RPL provides detection against sybil and rank attacks. However, the authors did not evaluate the impact of their approach in terms of performance, energy consumption, and performance overhead. A recent study in [98] presents a wormhole attack detection mechanism for the IoT routing protocol RPL. Their IDS placed at the border router (BR) as well as the host sensor nodes. They evaluated the impact of their IDs on the success rate for detecting the wormhole attack. Contiki OS simulation with three random typologies consists of 8, 16 and 24 sensor nodes among different run time. The result showed that the true positive rate was 96% with eight sensor nodes and decreases with the increase of sensor nodes to around 87%. The authors did not evaluate the impact of their IDs in terms of performance. They considered only the detection accuracy; furthermore, they detected only one specific type of attack rather than examine a range of attacks. Recently, a study in [99] proposed a hybrid IDS target the sinkhole and cloneID attacks and evaluated their ID impact in terms of performance and detection. The study was an extension of SVELTE and focused on improving the detection rate of false positive alarms. The authors claimed that the detection rate was 100%. However, the figures show that a detection rate decrease followed the increase in sensor nodes. Furthermore, the authors identified that the energy and power consumption was higher than the SVELTE. Currently, a study in [100] proposes IDS against a blackhole attack on routing protocol RPL. The authors aimed to improve detection efficiency by analysing only the suspected node rather than exploring all the nodes traffic as watchdog approaches do. However, the study limited the evaluation to address only the accuracy of their IDs.

Unlike intrusion detection on the Internet of Things, the authors of [101] implemented an intrusion detection framework and architecture that was an RPL-based IIoT that used genetic programming. Their detection approach targets two kinds of attack: in the version number and hello flood attacks, and they simulate the network in the Contiki OS. Their detection system shows that for the flood attack, 96.08% and 99.83% are the worst and the most accurate, respectively. The values were obtained by collecting data in the 500 ms and 5000 consecutive ms periods. The attack version number showed a performance closer to it in flood attack in different periods. The best and worst accuracy obtained with time intervals was 4000 ms (99.42%) and 3000 ms (97.97%), respectively. Although they investigated that genetic programming achieved high accuracy and low false positive in detecting intrusions, they restricted their investigation to only detection accuracy rather than examining the impact of their intrusion mechanism on performance such as energy and memory consumption. Table 3 shows the classification of intrusion detection systems for IoT network layer.

Another study in [102] implemented a detection mechanism for an industrial control system called HAMIDS, a hierarchical monitoring intrusion detection system. They employ a behaviour detection approach to the SCADA water treatment system application. The most important module is the Bro IDS sensor, which works on receiving data from the store in logs containing various network protocols such as UDP, TCP, Address Resolution Protocol (ARP) and EtherNet/IP. These logs are collected in the HAMIDS framework, then the components and results of IDS upon detecting threats from the raw records. The results show that the HAMIDS was able to detect outsider attacks with zero false positives. However, HAMID was still vulnerable to insider attacks such as DoS and MITM attacks level 0 specific threats to SCADA in realistic ICS such as Reboot Ethernet and CPU crashes. In the industrial IoT ecosystems, communication models among the devices are crucial for employing security such as anomalies detection and possible cyber-attacks. A study in [103] proposes a deep packet inspection based on discrete-time Markova chain models (DTMC) to four types of industrial networks datasets: energy management system, large-scale water treatment, small-scale water testbed and an electrical substation. The study evaluates the DTMC model’s impact on the industrial network’s datasets among various industrial communication protocols such as DNP3, EtherNet/IP and Modbus/TCP. While testing the data, the DTMC model’s potential was classified as unknown state, unknown transition and anomalous probability. The results show that the Energy System Monitor (ESM) dataset did not detect any unknown probabilities for two channels out of six. While for one of the channels, it observed a high number of transitions and states 248 and 479, respectively. In contrast, the power generation testbed dataset (PGT) did not observe any unknown probabilities and states (for the single state transitions). Afterwards, the maximum number of unknown probabilities 5 and unknown transitions 15 were observed. While the Water Plant Testbed (WPT) dataset had the highest number of unknown probabilities, 111 and 17.3 on average were of unknown transition. It is clear that more investigation is needed due to the high number of unknown transactions and states observed in all three datasets. It is clear from Table 3 that most studies concentrate mainly on insider attacks, while outsider attacks from the Internet must be studied and examined. As can be shown, attacks above the network layer have not been thoroughly studied. This clearly demonstrates that the transport and application layers of the Internet of Things will be vulnerable to attack, and IoT mentioned in the news because of DDoS attacks. For example, in 2016, the DNS provider that supported the internet services and platforms, including PayPal, VISA and Twitter, was attacked by DDoS through the vulnerabilities of IoT devices such as IP cameras, Printers and residential gateways that were infected by malware named Miria [8]. Another issue regarding IoT security research that the majority of authors keep their implementations’ source codes private. It will benefit the field of IoT security research if researchers share their implementation with the public.

### 7.3. Transport and Application Layer Security Solutions

The authors of [104,105] presented a security analysis between MQTT and CoAP with a specific focus on the transport protocol utilised MQTT with TLS and CoAP with DTLS. Furthermore, the comparative examination took into account security modes such as Raw Public Key, Certificates and Pre-Shared Key. The analysis shows that MQTT does not support RPK. However, it acts as a varied security alternative to lightweight and PSKs certificates. Nevertheless, the old certificate-based encryption and authentication offers the top level of security. Moreover, the use of certificates can make the HTTP more secure under different types of attack, as has already been proven. The authors of [106] applied RSA cryptography on sensing devices by using particular trusted-platform modules (TPM). They evaluated their system in terms of latency, energy consumption and memory based on a DTLS cipher suite TLS-RSA-with-AES-128-CBC-SHA. Further to this [107], the same proposal was described and further evaluated using an experiment in wireless sensor networks (WSN). Another study in [108] proposes an authentication security scheme for the transport layer using the ECC algorithm. They present DTLS implementation in the context of system architecture to achieve a low overhead and high interoperability. However, the results show that the handshake mechanism consumes higher energy because of the ECC algorithm’s complex computation. In contrast, the authors of [109] proposed an integrated DTLS with CoAP for IoT called Lithe. The authors propose a novel DTLS header compression scheme that aims to significantly reduce the header overhead of DTLS leveraging the 6LoWPAN standard. The authors evaluate their scheme based on cipher suite-based TLS-PSK-WITH-AES-128-CCM to reduce energy consumption and the round trip time using Contiki OS. The results show that Lithe is more efficient in many aspects than basic plain CoAP/DTLS. However, the proposed scheme lacks security against the DoS attack due to the DTLS cookie exchange scheme, which is insufficient to handle this type of attack, as the authors of [29] reported employing DTLS is a security solution that could only serve the security issues between UDP running on different endpoints. Still, it cannot protect the IP header when packets are transmitted through the access network layer of IoT objects through the internet. Nevertheless, they employed the DTLS protocol to reduce the number of transition bytes rather than secure the transmission through the IoT devices. In a similar study to Lithe, the authors of [110] present a lightweight DTLS for IoT called E-Lithe. They customise the DTLS packet to reduce energy consumption and execution time to reduce the DTLS computation overhead. The results show that energy and response time’s performance was reduced slightly more than in [109] study. It is essential to provide integrity and authentication security for IoT applications. The authors of [111] proposed identity-based lightweight encryption and a Diffie–Hellman encryption scheme for a smart home without using a digital certificate, in which case the public keys are only identity strings. This scheme is divided through the encryption process into data encryption and key encryption to gain more efficiency and reduce communication costs. The results show that the data ciphertext is transmitted several times without assigning the key ciphertext. Likewise, the performance analysis shows that the combination scheme of Identity-Based Encryption (IBE) and Diffie–Hellman have reduced the communication overhead by nearly one-third. It performs better than the symmetric IBE scheme in speeding up the encryption operations. In comparison, authors in [112] propose “integrity security” for Smart Home applications based on the CoAP protocol. They aim to add the optimal hash function to the CoAP protocol. They implemented and evaluated their study using Contiki. The result shows that the secure Hash Algorithm (SHA224) is the most optimal algorithm with CoAP when optimising power consumption and time. While the hash function algorithms do not intervene in the encryption/authentication. In the smart-home system terminology, data confidentiality is crucial due to many data transactions in the smart-home system. A similar study in [113] proposes a new architecture to secure CoAP, a smart-home application based on cryptography algorithms, based on AES and SHA224, instead of the traditional security for CoAP, which is based on DTLS. The study provides data confidentiality and integrity considering the constrained IoT device’s restriction in energy and execution time. However, the study does not compare their results with the traditional implementation of CoAP with DTLS. It appears that much research into securing CoAP protocol based on DTLS has been proposed, designed and implemented. Another study in [114] proposes secure CoAPs communication between the IoT devices in 6LoWPAN and smartphones. They evaluated the performance of the DTLS using the ECC. The use of complex computation when performing the ECC algorithm led to higher energy consumption.

In conclusion, considering the shortcomings, threats, cryptography-based security solutions and published research, there are still many problems to investigate in order to achieve a secure system, as highlighted in Table 4. There is a need to propose lightweight security algorithms by optimising algorithms such as DTLS and AES to support resource-constrained devices. Another issue mentioned above regarding IoT security research is that most authors keep their implementations’ source codes private. It will benefit the field of IoT security research if researchers share their implementation with the public.

## 8. Open Research Challenges

Although other studies might not be mentioned, this study provides a comprehensive overview of IoT security included in IoT layers, attacks, solutions, limitations and countermeasure studies in the literature. There are still many issues to be studied and solved with future research. As such, the following is some potential work that could be investigated in the future:

- The IoT devices have a limited processing capability, memory and storage, which they need to operate at low power. Security methods that demand heavy encryption are not suitable for constrained devices due to the complexity of encryption and decryption operations for transmitting data quickly and securely. Thus, lightweight encryption algorithms are needed for constrained devices, including actuators and sensors. Communication between these devices must be protected and provide integrity and confidentiality using hash functions and the AES.
- The implementation of the IDS in IoT networks presents new challenges, as it generates a large number of false alerts. It is a challenge to provide real-time IoT-IDSs and extend the range of attacks detection and consider the impact of the IDS on IoT devices’ performance regarding overhead, energy consumption and accuracy.
- The new era of Industry 4.0 and industrial IoT requires designing a novel intrusion detection methodology to guarantee the protection of connected systems and provided services.
- Prevention mechanisms for specific attacks to the IIoT environment such as smart grid, transportation, smart industry etc., need further development.
- Developing a lightweight security scheme for the smart grid application based on less computation algorithm suitable for constrained devices.

## 9. Conclusions

The new technology of IoT is enabling physical network connectivity and computing capability of sensors and control system to generate, exchange and consume data with minimal human interaction. This survey has presented various security threats at different IoT layers, including security challenges and solutions regarding the end-to-end IoT environment. It has covered the security issues related to the network layer, middleware layer, communication protocols and application layer. Further, it has provided a critical analysis of existing IoT solutions based on different security mechanisms, including cryptography and IDSs. The state-of-the-art IoT security has also been discussed with some of the future research directions to enhance IoT security levels. This survey is expected to become a roadmap toward security enhancement for IoT industrial applications.

## Figures and Tables

**Table 1:** Comparison of relevant surveys.

Ref	Year	Objective	Comparison with Our Survey
[16]	2019	Highlights the relevant security solutions regarding the middle layer and mobile device	This survey highlights classifications of security attacks and countermeasures in respect to each layer of IoT/IIoT layers
[17]	2015	Analyses and discusses the security issues of the IoT layers	This survey provides a critical evaluation of the existing security issues and countermeasures regarding IoT/IIoT layers
[18]	2018	Highlights the security and issues of the IoT layers	This survey presents a comprehensive evaluation of the security issues, attacks and solutions with respect to the IoT/IIoT layers
[19]	2019	Analyses the possible threats to the communication protocols on each layer	This study highlights the IIoT system integrity along with a case study discussing the existing security solutions for the important industrial IoT application of smart grids
[20]	2015	Investigates the security and issues related to the MAC layer and physical layer, IEEE802.15.4	This survey highlights classifications of security attacks and countermeasures with respect to each IoT/IIoT layer
[21]	2018	Reviews the most used cryptography algorithms on the IoT constrained deceives	This survey overviews various security solutions approaches mapped to each IoT/IIoT layer
[22]	2017	Details the various types of IoT application and discuss security and privacy in the IoT	This survey highlights classifications of security attacks and countermeasures mapped to each IoT/IIoT layer
[23]	2018	Highlights an appropriate cryptography algorithm covering the aspects of energy consumption and execution time	This survey reviews various security solutions approaches mapped to each layer of IoT/IIoT layer with their corresponding countermeasure proposed in the literature
[24]	2019	Examine and compare several lightweight algorithms that implemented on different tools and software	This work presents lightweight and non-lightweight algorithms and their possible solutions proposed in the literature.
[25]	2017	Compare possible attacks on IoT in the aspects of communication and application architecture	This survey highlights a comprehensive evaluation of the security issues, attacks and solutions with respect to each IoT/IIoT layer
[26]	2017	Analyse the security issues in IoT application based on smart cities	This survey reviews various security solutions approaches mapped to each layer of IoT layers with their corresponding countermeasure proposed in the literature
[27]	2018	Discuss the security and issues related to data privacy, trust and identification and authentication: uses smart manufacturing as case study	This study highlights system integrity of each layer of IoT/IIoT along with a case study discussing the existing security solutions for the most important industrial IoT application, the smart grid
[28]	2019	Highlights the vulnerabilities and solutions related to the privacy and routing attacks, mainly DoS	This survey highlight classifications of security attacks and countermeasures mapped to each layer of IoT/IIoT layers
[29]	2018	Highlights the characteristic of IoT and discuss the D/DoS attacks targeting the network and MAC layer	This survey highlights a comprehensive evaluation of the security issues, attacks and solutions with respect to IoT/IIoT layers
[30]	2019	Highlights and discusses the recent studies in the IoT security and issues from 2016 to 2018	This survey highlights a comprehensive evaluation of the security issues, attacks and solutions with respect to IoT/IIoT layers
Our Contribution	-	-	IIoT system integrity has been highlighted along with a case study discussing the existing security solutions for for an important IoT application, the smart grid; the taxonomy of IoT layers, attacks, and security countermeasures have been presented; provides a critical analysis of the practical implementation regarding IoT/IIoT; highlights several simulation tools and operating systems (OS) used to investigate security solutions; discusses potential future research directions

**Table 2:** IoT stack based on the standardised security solutions.

IoT Layers	Protocols	Security Solution
Application layer	CoAP, MQTT	CoAPs, MQTTs
Transport layer	UDP, TCP	DTLS, TLS
Network layer	IPv6, RPL	IDS, Secure RPL, IPsec
Perception layer	IEEE 802.14.5	IEEE 802.24.5 security

**Table 3:** Classification of intrusion detection systems for IoT network layer.

Ref	Detection Technique	Security Threats	Validation	Advantages	Disadvantages
[89]	Lightweight IDS	DoS attack	Simulation (Qualnet)	Evaluates the energy consumption.	Does not evaluate the accuracy.Use only one metric and one attack.
[90]	Centralised	Malicious attacks	RaspberryPi	High accuracy rate. Much faster than Wu-Manber.	High overhead.
[91]	Distributed	Snikhole attack	-	High accuracy rate.	High false-positive.Does not address the impact of their IDS on energy consumption.
[92]	Hybrid	Snikhole and Selective forwarding attacks	Cooja-ContikiOS	High accuracy rate and Low overhead.	High false alarm rate 38%
[93]	Centralised	Wormhole attack	Cooja-ContikiOS	High true-positive when the network size small.	the true-positive significantly decreases by the increase of network size.Evaluates only one metric and one attack.
[94]	Distributed	Repair and rank attack	Not available	Effectively detects rank and repair attacks.	Does not validate their approach on a simulation.
[95]	Centralised	Rank local repair, and sinkhole attacks	Cooja-ContikiOS	High detection accuracy Low false-positive.	Slight overhead.
[96]	-	Authentication key exchange and privacy	AVISSPA	Reduces latency and packet delay.	-
[97]	Distributed	Sybil and rank attack	Cooja-ContikiOS	Capable of detecting sybil and rank attacks.	Does not evaluate the impact of their approach in terms of performance.
[98]	Hybrid	Wormhole attack	Cooja-ContikiOS	High detection rate sufficient for constrained resources.	Detects only one attack.
[99]	Hybrid	Sinkhole-CloneID attacks	Cooja-ContikiOS	High detection rate.	High energy and power consumption.
[100]	Anomaly	Blackhole attack	Cooja-ContikiOS	Improves detection rate.	Packet delivery ratio decreases when the network size increases.
[101]	centralised	Version number and hello flood attacks	Cooja-ContikiOS	High accuracy detection.Low false-positive alarms	Evaluates only one metric which detection accuracy.Does not evaluate the impact of the IDs on performance.
[102]	Network behaviour anomaly	Outsider attacks ARP posing and MITM attacks	NS3 and Python script	Detects outsider attacks with zero false-positive.	Not able to detect insider attacks.
[103]	Anomaly	Malicious traffic	Discrete Markov Chain models (DTMC)	Evaluates the DTMC on different industrial datasets.	High number of unknown transactions and states.

**Table 4:** Classification of IoT security solution for transport and application.

Ref	Security Objective	Proposed Solution	Simulation/Testbed	Advantages	Disadvantages
[104,105]	Security analysis for MQTT-TLS and CoAP with DTLS	Authentication key exchange and security privacy	-	Compares the security mechanisms for MQTT and CoAP protocols.	
[106]	Provides message integrity and confidentiality with low overhead and latency	DTLS based on x.509 certificate containing RSA keys	TinyOS	Provides message integrity and confidentiality with energy efficiency	-
[107]	Provides data confidentiality, integrity with low overhead and high interoperability	integrated DTLS handshake and RSA key, and DTLS with ECC	Real IoT system	-	-
[108]	Provides authentication security and secure communication	DTLS with ECC public key authentication	TinyOS		Consumes higher energy due to the complexity computation of ECC algorithm
[109]	Reduces the energy consumption for the integration of CoAP with DTLS	Using tinyOS DTLS based on pre-shared keys	Cooja-ContikiOS	significantly reduces the overhead	Does not compromise end-to-end security
[110]	Lightweight security against DoS attack	Uses Trusted Third Party between the constrained node and CoAP server	Cooja-ContikOS	Reduce the energy and respond time	-
[111]	Lightweight encryption for smart-home based on public key	Cryptography solution based on stateful Diffie Hellman key and identity-based encryption scheme	-	Reduce the computational cost of encryption implementation	-
[112]	Message integrity security for smart-home application	integrated hash functions with CoAP protocol	Cooja-ContikiOS	Provides integrity protection	Does not provide data confidentiality. In the terminology of smart-home system, the data transactions being leaked
[113]	Data confidentiality, integrity, and authentication for smart-home application	Cryptography solution based on integrated CoAP-AES and CoAP-ShA2	Cooja-ContikiOS	Provides data confidentiality, integrity, and authentication protection	Does not evaluate their solution performance with DTLS
[114]	end-to-end security for mobile devices	Integrated DTLS with ECC authentication	TniyOS	-	-

## Data Availability

Not Applicable.

## References

[1] Girard M. (2020). Standards for Cybersecure IoT Devices: A Way Forward. JSTOR.

[2] Al-Rubaye S., Rodriguez J., Fragonara L.Z., Theron P., Tsourdos A. (2019). Unleash Narrowband Technologies for Industrial Internet of Things Services. IEEE Netw..

[3] Al-Rubaye S., Kadhum E., Ni Q., Anpalagan A. (2019). Industrial Internet of Things Driven by SDN Platform for Smart Grid Resiliency. IEEE Internet Things J..

[4] Wan J., Tang S., Shu Z., Li D., Wang S., Imran M., Vasilakos A.V. (2016). Software-defined industrial internet of things in the context of industry 4.0. IEEE Sens. J..

[5] Sengupta J., Ruj S., Bit S.D. (2020). A comprehensive survey on attacks, security issues and blockchain solutions for IoT and IIoT. J. Netw. Comput. Appl..

[6] Lee I., Lee K. (2015). The internet of things (iot): Applications, investments, and challenges for enterprises. Bus. Horizons.

[7] Hp News hp Study Reveals 70 Percent of Internet of Things Devices Vulnerable to Attack. https://www8.hp.com/us/en/hp-news/press-release.html?id=1744676.

[8] Flashpoint—Mirai Botnet Linked to Dyn DNS DDoS Attacks. https://www.flashpoint-intel.com/blog/cybercrime/mirai-botnet-linked-dyn-dns-ddos-attacks/.

[9] Agrawal M., Zhou J., Chang D. (2019). A survey on lightweight authenticated encryption and challenges for securing industrial IoT. Security and Privacy Trends in the Industrial Internet of Things.

[10] Zhang Y., Huang X. (2019). Security and privacy techniques for the industrial Internet of Things. Security and Privacy Trends in the Industrial Internet of Things.

[11] Sadeghi A.-R., Wachsmann C., Waidner M. Security and privacy challenges in industrial internet of things. Proceedings of the 52nd ACM/EDAC/IEEE Design Automation Conference (DAC).

[12] Li J.-Q., Yu F.R., Deng G., Luo C., Ming Z., Yan Q. (2017). Industrial internet: A survey on the enabling technologies, applications, and challenges. IEEE Commun. Surv. Tutor..

[13] Hoffman F. (2019). Industrial internet of things vulnerabilities and threats: What stakeholders need to consider. Issues Inf. Syst..

[14] Yu X., Guo H. A survey on IIoT security. Proceedings of the 2019 IEEE VTS Asia Pacific Wireless Communications Symposium (APWCS).

[15] Yadav G., Paul K. (2021). Architecture and Security of SCADA Systems: A Review. Int. J. Crit. Infrastruct. Prot..

[16] Sicari S., Rizzardi A., Grieco L.A., CoenPorisini A. (2015). Security, privacy and trust in internet of things: The road ahead. Comput. Netw..

[17] Jing Q., Vasilakos A.V., Wan J., Lu J., Qiu D. (2014). Security of the in-ternet of things: Perspectives and challenges. Wirel. Netw..

[18] Frustaci M., Pace P., Aloi G., Fortino G. (2017). Evaluating critical security issues of the iot world: Present and future challenges. IEEE Internet Things J..

[19] Grammatikis P.I.R., Sarigiannidis P.G., Moscholios I.D. (2019). Securing the internet of things: Challenges, threats and solutions. Internet Things.

[20] Granjal J., Monteiro E., Silva J.S. (2015). Security for the internet of things: A survey of existing protocols and open research issues. IEEE Commun. Surv. Tutor..

[21] Malina L., Hajny J., Fujdiak R., Hosek J. (2016). On perspective of security and privacy-preserving solutions in the internet of things. Comput. Netw..

[22] Maple C. (2017). Security and privacy in the internet of things. J. Cyber Policy.

[23] Sadeeq M.A., Zeebaree S.R., Qashi R., Ahmed S.H., Jacksi K. Internet of things security: A survey. Proceedings of the 2018 International Conference on Advanced Science and Engineering (ICOASE).

[24] Shah A., Engineer M. (2019). A survey of lightweight cryptographic algorithms for iot-based applications. Smart Innovations in Communication and Computational Sciences.

[25] Alaba F.A., Othman M., Hashem I.A.T., Alotaibi F. (2017). Internet of things security: A survey. J. Netw. Comput. Appl..

[26] Latif S., Zafar N.A. A survey of security and privacy issues in iot for smart cities. Proceedings of the 2017 Fifth International Conference on Aerospace Science & Engineering (ICASE).

[27] Sfar A.R., Natalizio E., Challal Y., Chtourou Z. (2018). A roadmap for security challenges in the internet of things. Digit. Commun. Netw..

[28] Hameed S., Khan F.I., Hameed B. (2019). Understanding security requirements and challenges in internet of things (iot): A review. J. Comput. Netw. Commun..

[29] Arıs A., Oktug S.F., Voigt T. (2018). Security of Internet of Things for a Reliable Internet of Services.

[30] Hassan W.H. (2019). Current research on internet of things (iot) security: A survey. Comput. Netw..

[31] Stouffer K., Tang C., Zimmerman T., Powell M., McCarthy J., Ogunyale T., Acierto L., Danley L. (2020). Protecting Information and System Integrity in Industrial Control Systems Environments: Cybersecurity for the Manufacturing Sector.

[32] Stouffer K., Tang C., Zimmerman T., Powell M., McCarthy J., Ogunyale T., Acierto L., Danley L. (2019). Detecting and Protecting against Data Integrity Attacks in Industrial Control Systems Environments: Cybersecurity for the Manufacturing Sector (Draft).

[33] Elyoenai E., Daniel R., Jairo O., Victor V., Luis T. (2012). Smart Grid Security. European Network and Information Security Agency (ENISA). https://www.enisa.europa.eu/publications/ENISA-smart-grid-security-recommendations.

[34] Sarenche R., Salmasizadeh M., Ameri M.H., Aref M.R. (2021). A secure and privacy-preserving protocol for holding double auctions in smart grid. Inf. Sci..

[35] Khan A.A., Kumar V., Ahmad M., Rana S. (2021). LAKAF: Lightweight authentication and key agreement framework for smart grid network. J. Syst. Archit..

[36] Abdallah A., Shen X.S. (2016). A lightweight lattice-based homomorphic privacy-preserving data aggregation scheme for smart grid. IEEE Trans. Smart Grid.

[37] Abbasinezhad-Mood D., Nikooghadam M. (2018). An anonymous ECC-based self-certified key distribution scheme for the smart grid. IEEE Trans. Ind. Electron..

[38] Grover H.S., Kumar D. (2020). Cryptanalysis and improvement of a three-factor user authentication scheme for smart grid environment. J. Reliab. Intell. Environ..

[39] Deng L., Gao R. (2021). Certificateless two-party authenticated key agreement scheme for smart grid. Inf. Sci..

[40] Chaudhry S.A., Alhakami H., Baz A., Al-Turjman F. (2020). Securing demand response management: A certificate-based access control in smart grid edge computing infrastructure. IEEE Access.

[41] Wang Z., Liu Y., Ma Z., Liu X., Ma J. (2012). LiPSG: Lightweight Privacy-Preserving Q-Learning-Based Energy Management for the IoT-Enabled Smart Grid. IEEE Internet Things J..

[42] Deogirikar J., Vidhate A. Security attacks in IoT: A survey. Proceedings of the 2017 International Conference on I-SMAC (IoT in Social, Mobile, Analytics and Cloud)(I-SMAC).

[43] Kumar S., Sahoo S., Mahapatra A., Swain A.K., Mahapatra K.K. Security enhancements to system on chip devices for IoT perception layer. Proceedings of the 2017 IEEE International Symposium on Nanoelectronic and Information Systems (iNIS).

[44] Mohite S., Kulkarni G., Sutar R., Mohite S. (2013). RFID security issues. Int. J. Eng. Res. Technol. (IJERT).

[45] Wallgren L., Raza S., Voigt T. (2013). Routing attacks and countermeasures in the RPL-based internet of things. Int. J. Distrib. Sens. Netw..

[46] Pongle P., Chavan G. A survey: Attacks on RPL and 6LoWPAN in IoT. Proceedings of the 2015 International Conference on Pervasive Computing (ICPC).

[47] Nawir M., Amir A., Yaakob N., Lynn O.B. Internet of Things (IoT): Taxonomy of security attacks. Proceedings of the 2016 3rd International Conference on Electronic Design (ICED).

[48] Abdul-Ghani H.A., Konstantas D., Mahyoub M. (2018). A comprehensive IoT attacks survey based on a building-blocked reference model. Int. J. Adv. Comput. Sci. Appl..

[49] Karlof C., Wagner D. (2003). Secure routing in wireless sensor networks: Attacks and countermeasures. Ad Hoc Netw..

[50] Gautam S., Malik A., Singh N., Kumar S. Recent Advances and Countermeasures Against Various Attacks in IoT Environment. Proceedings of the 2019 2nd International Conference on Signal Processing and Communication (ICSPC).

[51] Rizvi S., Kurtz A., Pfeffer J., Rizvi M. Securing the internet of things (iot): A security taxonomy for iot. Proceedings of the 2018 17th IEEE International Conference on Trust, Security and Privacy in Computing and Communications/12th IEEE International Conference on Big Data Science and Engineering (TrustCom/BigDataSE).

[52] Hari P.B., Singh S.N. Security issues in wireless sensor networks: Current research and challenges. Proceedings of the 2016 International Conference on Advances in Computing, Communication, & Automation (ICACCA)(Spring).

[53] Ali W., Dustgeer G., Awais M., Shah M.A. Iot based smart home: Security challenges, security requirements and solutions. Proceedings of the 2017 23rd International Conference on Automation and Computing (ICAC).

[54] Hossain M.M., Fotouhi M., Hasan R. Towards an analysis of security issues, challenges, and open problems in the internet of things. Proceedings of the 2015 IEEE World Congress on Services.

[55] Lin J., Yu W., Zhang N., Yang X., Zhang H., Zhao W. (2017). A survey on internet of things: Architecture, enabling technologies, security and privacy, and applications. IEEE Internet Things J..

[56] Vashi S., Ram J., Modi J., Verma S., Prakash C. Internet of things (iot): A vision, architectural elements, and security issues. Proceedings of the 2017 International Conference on I-SMAC (IoT in Social, Mobile, Analytics and Cloud)(I-SMAC).

[57] Liu X., Zhao M., Li S., Zhang F., Trappe W. (2017). A security framework for the internet of things in the future internet architecture. Future Internet.

[58] Andrea I., Chrysostomou C., Hadjichristofi G. Internet of things: Security vulnerabilities and challenges. Proceedings of the 2015 IEEE Symposium on Computers and Communication (ISCC).

[59] Yousuf T., Mahmoud R., Aloul F., Zualkernan I. (2015). Internet of things (iot) security: Current status, challenges and countermeasures. Int. J. Inf. Secur. Res. (IJISR).

[60] Dragomir D., Gheorghe L., Costea S., Radovici A. A survey on secure communication protocols for iot systems. Proceedings of the 2016 International Workshop on Secure Internet of Things (SIoT).

[61] Naoui S., Elhdhili M.E., Saidane L.A. Enhancing the security of the IoT LoraWAN architecture. Proceedings of the 2016 International Conference on Performance Evaluation and Modeling in Wired and Wireless Networks (PEMWN).

[62] Aras E., Ramachandran G.S., Lawrence P., Hughes D. Exploring the security vulnerabilities of LoRa. Proceedings of the 2017 3rd IEEE International Conference on Cybernetics (CYBCONF).

[63] Keoh S.L., Kumar S.S., Tschofenig H. (2014). Securing the internet of things: A standardization perspective. IEEE Internet Things J..

[64] Raza S., Duquennoy S., Voigt T., Roedig U. Demo abstract: Securing communication in 6lowpan with compressed ipsec. Proceedings of the 2011 International Conference on Distributed Computing in Sensor Systems and Workshops (DCOSS).

[65] Arfaoui A., Kribeche A., Senouci S.M., Hamdi M. Game-based adaptive remote access VPN for IoT: Application to e-Health. Proceedings of the IEEE Global Communications Conference (GLOBECOM).

[66] Khan M.A., Salah K. (2018). Iot security: Review, blockchain solutions, and open challenges. Future Gener. Comput. Syst..

[67] Assiri B., Almagwashi H. Iot security and privacy issues. Proceedings of the 2018 1st International Conference on Computer Applications Information Security (ICCAIS).

[68] Bilal M. (2017). A review of internet of things architecture, technologies and analysis smartphone-based attacks against 3d printers. arXiv.

[69] Airehrour D., Gutierrez J., Ray S.K. (2016). Secure routing for internet of things: A survey. J. Netw. Comput. Appl..

[70] Krishna B.S., Gnanasekaran T. A systematic study of security issues in internet-of-things (iot). Proceedings of the 2017 International Conference on I-SMAC (IoT in Social, Mobile, Analytics and Cloud)(I-SMAC).

[71] Singh D., Tripathi G., Jara A. Secure layers based architecture for internet of things. Proceedings of the 2015 IEEE 2nd World Forum on Internet of Things (WF-IoT).

[72] Benkhelifa E., Welsh T., Hamouda W. (2018). A critical review of practices and challenges in intrusion detection systems for iot: Toward universal and resilient systems. IEEE Commun. Surv. Tutor..

[73] Ammar M., Russello G., Crispo B. (2018). Internet of things: A survey on the security of iot frameworks. J. Inf. Secur. Appl..

[74] Chernyshev M., Baig Z., Bello O., Zeadally S. (2017). Internet of things (iot): Research, simulators, and testbeds. IEEE Internet Things J..

[75] Ani U.D., Watson J.M., Carr M., Cook A., Nurse J.R.C. (2020). A review of the use and utility of industrial network-based open source simulators: Functionality, security, and policy viewpoints. J. Def. Model. Simul..

[76] Al-Kashoash H. (2019). Congestion Control for 6LoWPAN Wireless Sensor Networks: Toward the Internet of Things.

[77] Varga A., Hornig R. An overview of the omnet++ simulation environment. Proceedings of the 1st International Conference on Simulation Tools and Techniques for Communications, Networks and Systems & Workshops, ICST (Institute for Computer Sciences, Social-Informatics).

[78] Choi H.-Y., Min S.-G., Han Y.-H., Park J., Kim H. Implementation and evaluation of proxy mobile ipv6 in ns-3 network simulator. Proceedings of the 2010 5th International Conference on Ubiquitous Information Technologies and Applications.

[79] Siraj S., Gupta A., Badgujar R. (2012). Network simulation tools survey. Int. J. Adv. Res. Comput. Commun. Eng..

[80] Queiroz C., Mahmood A., Tari Z. (2011). SCADASim—A framework for building SCADA simulations. IEEE Trans. Smart Grid.

[81] Zikria Y.B., Kim S.W., Hahm O., Afzal M.K., Aalsalem M.Y. (2019). Internet of things (iot) operating systems management: Opportunities, challenges, and solution. Sensors.

[82] Kumar G.S., Paul T. Safe contiki os: Type and memory safety for contiki os. Proceedings of the International Conference on Advances in Recent Technologies in Communication and Computing.

[83] Saadallah B., Lahmadi A., Festor O. (2012). Ccnx for Contiki: Implementation Details, Inria Nancy—Grand Est, LORIA—NSS: HAL-Inria.

[84] Samal N., Dalai P. (2018). A Performance survey of operating systems in iot environment. Int. J. Comput. Sci. Mob. Appl..

[85] Raspberry pi 4 Review: The New Gold Standard for Single-Board Computing-Tom’s hArdware Tom’s Hardware. USA. https://www.tomshardware.com/reviews/raspberry-pi-4-b,6193.html.

[86] Top 10 IoT Operating Systems For IoT Devices In 2021—Solace Infotech Pvt Ltd. USA. https://solaceinfotech.com/blog/top-10-iot-operating-systems-for-iot-devices-in-2021/.

[87] Raza S., Duquennoy S., Höglund J., Roedig U., Voigt T. (2014). Secure communication for the internet of things a comparison of link-layer security and ipsec for 6lowpan. Secur. Commun. Netw..

[88] Ndibanje B., Lee H.-J., Lee S.-G. (2014). Security analysis and improvements of authentication and access control in the internet of things. Sensors.

[89] Lee T.H., Wen C.H., Chang L.-H., Chiang H.S., Hsieh M.C. (2014). A lightweight intrusion detection scheme based on energy consumption analysis in 6lowpan. Advanced Technologies, Embedded and Multimedia for Human-Centric Computing.

[90] Oh D., Kim D., Ro W. (2014). A malicious pattern detection engine for embedded security systems in the internet of things. Sensors.

[91] Cervantes C., Poplade D., Nogueira M., Santos A. Detection of sinkhole attacks for supporting secure routing on 6lowpan for internet of things. Proceedings of the 2015 IFIP/IEEE International Symposium on Integrated Network Management (IM).

[92] Raza S., Wallgren L., Voigt T. (2013). Svelte: Real-time intrusion detection in the internet of things. Ad Hoc Netw..

[93] Deshmukh-Bhosale S., Sonavane S.S. (2019). A real-time intrusion detection system for wormhole attack in the rpl based internet of things. Procedia Manuf..

[94] Le A., Loo J., Luo Y., Lasebae A. Specification-based ids for securing rpl from topology attacks. Proceedings of the 2011 IFIP Wireless Days (WD).

[95] Le A., Loo J., Chai K., Aiash M. (2016). A specification-based ids for detecting attacks on rpl-based network topology. Information.

[96] Shin D., Sharma V., Kim J., Kwon S., You I. (2017). Secure and efficient protocol for route optimization in pmipv6-based smart home iot networks. IEEE Access.

[97] Airehrour D., Gutierrez J.A., Ray S.K. (2019). Sectrust-rpl: A secure trust-aware rpl routing protocol for internet of things. Future Gener. Comput. Syst..

[98] Pongle P., Chavan G. (2015). Real time intrusion and wormhole attack detection in internet of things. Int. J. Comput. Appl..

[99] Mirshahjafari S.M.H., Ghahfarokhi B.S. (2019). Sinkhole+ cloneid: A hybrid attack on rpl performance and detection method. Inf. Secur. J. Glob. Perspect..

[100] Patel H.B., Jinwala D.C. Blackhole detection in 6lowpan based internet of things: An anomaly based approach. Proceedings of the TENCON 2019-2019 IEEE Region 10 Conference (TENCON).

[101] Aydogan E., Yilmaz S., Sen S., Butun I., Forsström S., Gidlund M. A central intrusion detection system for rpl-based industrial internet of things. Proceedings of the 2019 15th IEEE International Workshop on Factory Communication Systems (WFCS).

[102] Ghaeini H.R., Tippenhauer N.O. Hamids: Hierarchical monitoring intrusion detection system for industrial control systems. Proceedings of the 2nd ACM Workshop on Cyber-Physical Systems Security and Privacy.

[103] Faisal M.A., Cardenas A.A., Wool A. (2019). Profiling communications in industrial ip networks: Model complexity and anomaly detection. Security and Privacy Trends in the Industrial Internet of Things.

[104] Zamfir S., Balan T., Iliescu I., Sandu F. A Security Analysis on Standard IoT Protocols. Proceedings of the 2016 International Conference on Applied and Theoretical Electricity (ICATE).

[105] Anthraper J.J., Kotak J. (2019). Security, Privacy and Forensic Concern of MQTT Protocol. SSRN Electron. J..

[106] Kothmayr T., Schmitt C., Hu W., Brunig M., Carle G. A dtls based end-to-end security architecture for the internet of things with two-way authentication. Proceedings of the 37th Annual IEEE Conference on Local Computer Networks-Workshops.

[107] Kothmayr T., Schmitt C., Hu W., Brunig M., Carle G. (2013). Dtls based security and two-way authentication for the internet of things. Ad Hoc Netw..

[108] Granjal J., Monteiro E., Silva J.S. End-to-end transport-layer security for internet-integrated sensing applications with mutual and delegated ecc public-key authentication. Proceedings of the 2013 IFIP Networking Conference.

[109] Raza S., Shafagh H., Hewage K., Hummen R., Voigt T. (2013). Lithe: Lightweight secure coap for the internet of things. IEEE Sens. J..

[110] Haroon A., Akram S., Shah M.A., Wahid A. E-lithe: A lightweight secure DTLS for IoT. Proceedings of the IEEE Vehicular Technology Conference.

[111] Salami S.A., Baek J., Salah K., Damiani E., Motivation A. Lightweight Encryption for Smart Home. Proceedings of the 2016 11th International Conference on Availability, Reliability and Security (ARES).

[112] Halabi D., Hamdan S., Almajali S. Enhance the security in smart home applications based on IOT-CoAP protocol. Proceedings of the 6th International Conference on Digital Information, Networking, and Wireless Communications, DINWC.

[113] Abosata N.R.A., Kemp A.H., Razavi M. Secure smart-home application based on iotcoap protocol. Proceedings of the 2019 Sixth International Conference on Internet of Things: Systems, Management and Security (IOTSMS).

[114] Granjal J., Monteiro E. End-to-end transparent transport-layer security for internet-integrated mobile sensing devices. Proceedings of the 2016 IFIP Networking Conference (IFIP Networking) and Workshops.

